# CDMBE: A Case Description Model Based on Evidence

**DOI:** 10.1155/2015/470818

**Published:** 2015-09-01

**Authors:** Jianlin Zhu, Xiaoping Yang, Jing Zhou

**Affiliations:** ^1^Information School, Renmin University of China, Beijing 100872, China; ^2^Hebei Finance University, Baoding 071051, China

## Abstract

By combining the advantages of argument map and Bayesian network, a case description model based on evidence (CDMBE), which is suitable to continental law system, is proposed to describe the criminal cases. The logic of the model adopts the credibility logical reason and gets evidence-based reasoning quantitatively based on evidences. In order to consist with practical inference rules, five types of relationship and a set of rules are defined to calculate the credibility of assumptions based on the credibility and supportability of the related evidences. Experiments show that the model can get users' ideas into a figure and the results calculated from CDMBE are in line with those from Bayesian model.

## 1. Introduction

So far, there are many classical models in legal argument domain, such as argument graph models of informal logic, argument map, and Bayesian network. Argument map has two stages, assumption recognition and assumption proof. User recognizes the assumptions according to the case and evaluates the possibility of the assumptions by evidences [1]. Wigmore chart is one of the argument maps [2], by which its user can analyze evidences and assumptions qualitatively and reasons out the authenticity of the final assumption. Wigmore chart includes supporting evidences and opposing evidences. It can help the user get his ideas into a figure. But it has no calculability. On the other hand, Bayesian network is a kind of probabilistic graphical model and it is suitable to reason the legal argument process. It describes the relationships between evidences and assumptions as probabilities and can realize quantitative reasoning. However, it requires that the user have a lot of background knowledge of mathematics, and it is not suitable to get the user's ideas into a figure [3].

The above studies are suitable for common law system. However, Chinese law system belongs to continental law system; that is, a judgment must be based on some legal provisions. Mighui Xiong, a professor of Sun Yat-sen University of China, a member of the International Association for the Artificial Intelligence and Law (IAAIL), thinks that traditional logic cannot provide logical defense for legal argumentation, but the informal argument can, which introduces coherence, adequacy, and acceptability to nonmonotonic logic to make legal arguments more reasonable [4–8]. In particular, a legal conclusion is acceptable in some places, but it is not always acceptable in other places. Even in the same place, it is not always acceptable in different ages. And all of the legal conclusions are adaptable, falsifiable, and defeasible. For combining the advantages of traditional logic and informal logic, we proposed a case description model based on evidence (called CDMBE later), which can get users' ideas into a figure, can clear up their thoughts, and has a strict calculability. More importantly, the premises and conclusions in the model are all open, which makes the model plausible and defeasible.

## 2. Related Work

The literatures about argument map can be traced to  1931 in [9]. Wigmore firstly used the concept. After that, many scholars did extending researches. The argumentation logic belongs to defeasible logic but is named differently [10]. Toulmin [11] and Verheij [12] called it defeasible argument; Hage [13] and Prakken [14, 15] called it “rule.” Pollock [16] used “reasoning rule” instead of it, while Walton [17] described it as “planning system.” The argumentation relationship is composed of a series of connected inferences, which make up an argument map [16]. Thus an argument map, like Wigmore chart [9], contains evidences as premises and one final assumption as the conclusion. Argument map can get its user's ideas into a figure and clear up his thought, but it is not a quantitative reasoning model.

Statistics method in information technology is usually used in legal reasoning. However, it has incurred the “argumentation about probability” [18] in the last 30 years. During this era, “People versus Collins” was a typical case for using a statistics method to solve an evidence-based reasoning problem. In 1977, it had a great influence on renewing interest in probability method that Lempert applied Bayesian network to evidence reasoning [19]. After that, scholars did a lot of researches on Bayesian method, for example, Taroni et al. [20], Dawid [21], and Jackson et al. [22]. Bayesian network can represent the relationships between evidences and assumptions in probabilities, which simplify the reasoning process [23] and can be used for quantitative reasoning [24]. However, it requires that the user have a lot of background knowledge of mathematics, and it is not suitable to get the user's ideas into a figure.

## 3. Case Description Model Based on Evidence

CDMBE combines the intuition of Wigmore chart with the calculability of Bayesian network, by which the final assumption's credibility can be figured out by 5 kinds of syntagmatic relationships between evidences and intermediate assumptions based on credibility and supportability. CDMBE has a good description capability that it can describe any legal case. And it has a strict calculability that the reasoning results are reasonable when the credibility and supportabilities are correctly set by a user. CDMBE can be used as a tool of case analysis and knowledge storage so that the knowledge can be used as a data source of legal data mining.

### 3.1. Evidence

Evidence means any fact that can prove an assumption is reasonable. It comes from a series of legal procedures, for example, collecting, examining, and verifying materials. Chinese procedural law specifies 7 kinds of evidences: physical evidence, witness testimony, statement of victim, expert conclusion, investigation records, audiovisual material, and other evidences. In order to represent accurately knowledge of legal cases, we introduce 7 kinds of evidences into CDMBE and define two characters, credibility and supportability.

Physical evidence is an object or a trace, which can prove the truth of a case by its external features, attributes, and existing form. Physical evidence is indirect and dependent, which must be used with the other evidence to confirm one another. For example, fingerprints left at the scene must be the same as the defendant's fingerprints to prove the defendant was once in the scene. In CDMBE, the rectangle is used to represent physical evidence.

Witness testimony refers to the witness's statement about what he perceived in the case. The effectiveness of the witness testimony can be estimated by the qualification of the witness, authenticity, and objectivity. In CDMBE, punched tape is used to represent witness testimony.

Statement of victim refers to the statement made by a victim. The effectiveness of the statement of victim can be estimated by the following aspects: source, rationality, relationship between the victim and the defendant, ability and character of proof, conflicts with other evidences, and so forth. In CDMBE, parallelogram is used to represent the statement of victim.

Expert conclusion is a conclusive report made by commissioned appraiser after checking, analyzing, and judging a professional problem with specialized knowledge and modern technology. The following aspects need be considered: whether the appraiser is qualified, whether the proof ability of the identified material is sufficient, whether the identification method is scientific, whether the appraiser is affected by other events, and whether the conclusion conflicts with other evidences. In CDMBE, stored data is used to represent expert conclusion.

Investigation records refer to the investigation records about the crime scene, objects, and bodies made by professional investigators according to their privileges and legal procedures. In general, investigation records are objective and strong proofs. The effectiveness of the investigation records can be estimated by the following aspects: whether they are made according to legal procedures, whether the content is objective, complete, and accurate, and whether the facts in the recording can be confirmed with other evidences. In CDMBE, internal storage is used to represent investigation records.

Audiovisual material means multimedia information stored in tape, CD, computer, and so forth. In CDMBE, card is used to represent this kind of material.

Evidence that does not belong to the above types can be classified into other evidences. In CDMBE, document is used to represent other evidences.

### 3.2. Assumption

Assumption is one of the human thinking methods. It is a speculative statement on an unknown fact, which begins with facts and reasoning by scientific methods.

The assumptions in CDMBE include amount of intermediate assumptions and a final assumption, the conclusion. Like evidences, assumptions also have credibility and supportability. But its credibility is calculated from the lower level evidences and assumptions.

### 3.3. The Testimonial Power of Evidence

The testimonial power comes from Law of Evidence. Since it is not defined in Chinese law, its concept is not unified [25–27]. In this paper, it is defined as the adopted qualification of evidence, and it can be calculated by credibility and supportability in the model.

In Law of Evidence, the testimonial power has three characters: objectivity, relevance, and legality [28]. The objectivity means that evidence has objective authenticity. The relevance means evidence has actual connection with the case. And the legality means that the procedures of collecting, showing, and checking are legal.

The credibility is a character of evidence and assumption. And it is a quantitative description about the objectivity and legality of evidence or assumption. The supportability is a quantitative description about the relationship between assumption and its lower level evidences or assumptions. It is represented as a line with an arrow in CDMBE. The credibility is a decimal between 0 and 1; the supportability is a decimal between −1 and 1. When positive, the evidence has confirmed effect on the conclusion. When negative, the evidence has falsified effect on the conclusion.

If the probability of Tom holding the murderous weapon at the crime scene is 0.9 (in other words, the credibility of the intermediate assumption is 0.9, and its supportability for Tom killing someone is 0.8), a direct way to calculate the testimonial power is to multiply credibility and supportability. But when credibility and supportability are both small (e.g., they are both 0.4), the testimonial power descends too fast. In order to mitigate the trend, the formula is defined as follows.

Suppose *i* is an evidence or an assumption, its credibility is *C*_*i*_, and its supportability for the high level assumption is *S*_*i*_; then its testimonial power *P*_*i*_ can be defined as

(1) $$\begin{array}{c} P_{i}=\frac{C_{i}*S_{i}}{C_{i}*S_{i}+C_{i}*\left(1-S_{i}\right)+S_{i}*\left(1-C_{i}\right)}. \end{array}$$

### 3.4. Syntagmatic Relationship Calculation

The credibility of an assumption can be calculated by the testimonial powers of evidences or assumptions in lower level. However, the relationships among evidences and assumptions are complicated, which are classified as conjunction, recombination, aggregation, reinforcement, and coupling in Law of Evidence [28]. In this section, the relationships are transformed into formulas and introduced into CDMBE.

#### 3.4.1. Conjunction

Conjunction means that all the constitutive requirements of an assumption must be proved in order to prove the assumption. For example, to prove an assumption that Tom was convicted of killing someone, the four constitutive requirements must be proved; that is, the subject of crime, Tom, is a person with full criminal responsibilities, the subjectivity is on purpose, the object of crime is a person's life, and the objectivity is the fact that Tom took the person's life. Only when the four constitutive requirements are proved correct, the assumption is true.

Suppose *h* is an assumption, *P*_1_, *P*_2_,…, *P*_*n*_ are the testimonial powers of evidences and assumptions in lower level, and their relationship is conjunction; then *h*'s credibility *C*_*h*_ can be defined as

(2) $$\begin{array}{c} C_{h}=\min\left(P_{1},P_{2},\ldots ,P_{n}\right). \end{array}$$

#### 3.4.2. Recombination

Recombination means that an assumption can only be proved true when all required evidences exist at the same time. For example, it needs to be proved that Tom has a chance to kill Jerry at Jerry's home. Evidence 1 is that Jerry died at his home. Evidence 2 is that Tom was at Jerry's home when Jerry died. Recombination is similar to conjunction, and in CDMBE they have the same formula (formula (2)), but recombination is usually used in the combination relationship of evidences, while conjunction is usually used in the combination relationship of constitutive requirements.

#### 3.4.3. Aggregation

When two independent evidences support one assumption and make it stricter, the two evidences have aggregation relationship. For example, evidence 1 shows Tom arrived at Jerry's home at 8:20 am, evidence 2 shows that Tom left Jerry's home at 8:35 am, and then it can be firmly proved that Tom is at Jerry's home at 8:30 am.

By analyzing some cases, we think that the aggregation result should be stronger than the strongest testimonial power when the evidences' testimonial powers are all strong, while the aggregation result should be weaker than the weakest one when the evidences' testimonial powers are all weak, and the aggregation result should be between the weakest and the strongest one when some testimonial powers are strong and others are weak.

Suppose *h* is an assumption, *P*_1_, *P*_2_,…, *P*_*n*_ are the descending order of the testimonial powers of evidences and assumptions in lower level, and their relationship is aggregation; then *h*'s credibility *C*_*h*_ can be defined as

(3) $$\begin{array}{c} a_{1}=P_{1},\\ \underset{2\leq i\leq n}{a_{i}}=\left(2-P_{i}*a_{i-1}\right)*P_{i}*a_{i-1},\;\left(P_{i},a_{i-1}\geq 0.7\right),\\ \underset{2\leq i\leq n}{a_{i}}=\left(1+P_{i}*a_{i-1}\right)*\frac{\left(P_{i}+a_{i-1}\right)}{2},\;\left(0.4\leq P_{i},a_{i-1}<0.7\right),\\ \underset{2\leq i\leq n}{a_{i}}=\left(1-P_{i}*a_{i-1}\right)*\frac{\left(P_{i}+a_{i-1}\right)}{2},\;\left(P_{i},a_{i-1}\leq 0.4\right),\\ \underset{2\leq i\leq n}{a_{i}}=a_{i-1}*\left(1-P_{i}*s_{b_{i}}\right),\;\left(a_{i-1}\geq 0.7,P_{i}<0.4\right),\\ \underset{2\leq i\leq n}{a_{i}}=\left(1-P_{i}*P_{i}\right)*a_{i-1},\;\left(P_{i}\geq 0.7,a_{i-1}<0.4\right),\\ \underset{2\leq i\leq n}{a_{i}}=a_{i-1},\;\left(\text{others}\right),\\ C_{h}=a_{n}. \end{array}$$

#### 3.4.4. Reinforcement

The concept of reinforcement is from common law system. It is defined as a relationship between supplemental evidences and the chief evidence, which can strengthen the chief evidence's testimonial power.

Suppose *h* is an assumption, MP is the testimonial power of the chief evidence, *P*_1_, *P*_2_,…, *P*_*n*_ are the descending order of the testimonial powers of supplemental evidences, and their relationship is reinforcement; then *h*'s credibility *C*_*h*_ can be defined as

(4) $$\begin{array}{c} a_{1}=MP,\\ a_{i}=\left(1+\frac{\left(1-a_{i-1}\right)*P_{i-1}}{2}\right)*a_{i-1},\;\left(P_{i-1}\geq 0.7,2\leq i\leq n\right),\\ a_{i}=\left(1+\frac{\left(1-a_{i-1}\right)*P_{i-1}}{3}\right)*a_{i-1},\;\left(0.4<P_{i-1}<0.7,2\leq i\leq n\right),\\ a_{i}=\left(1+\frac{\left(1-a_{i-1}\right)*P_{i-1}}{2}\right)*a_{i-1},\;\left(a_{i-1}\leq 0.4,P_{i}\leq 0.4,2\leq i\leq n\right),\\ a_{i}=a_{i-1},\;\left(a_{i-1}>0.4,P_{i-1}<0.4,2\leq i\leq n\right),\\ C_{h}=a_{n}. \end{array}$$

#### 3.4.5. Coupling

Coupling means a chain of reasoning. That is, when an assumption is proved by another assumption, their relationship is coupling. The coupling's credibility can be calculated by formula (1) because it describes a multilayer relationship.

## 4. Experiments

The case in Agatha Christie's novel “Witness for the Prosecution” is selected as the experimental data. In the section, authors describe the facts, analyze the case, simulate the court trial process, reason the judgment by CDMBE, and compare the analysis results with the Bayesian Network model built by Norman Fenton.

### 4.1. Top-Level Graph Based on Constitutive Requirements

When drawing a CDMBE graph, a top-down process must be followed, which means that a top-level graph must be drawn firstly and then unfolded. The top-level graph is composed of a final assumption and several constitutive requirements.

A case generally has 4 common requirements: the subject refers to the person who implements the criminal act, the object refers to a social relationship, like political or economic relationship, which is protected by law and violated by the criminal act, the subjectivity refers to the subjective attitude of defendant when he or she commits a crime, and the objectivity refers to the criminal act [28].

### 4.2. The CDMBE Graph of “Witness for the Prosecution”

**Figure 1:**
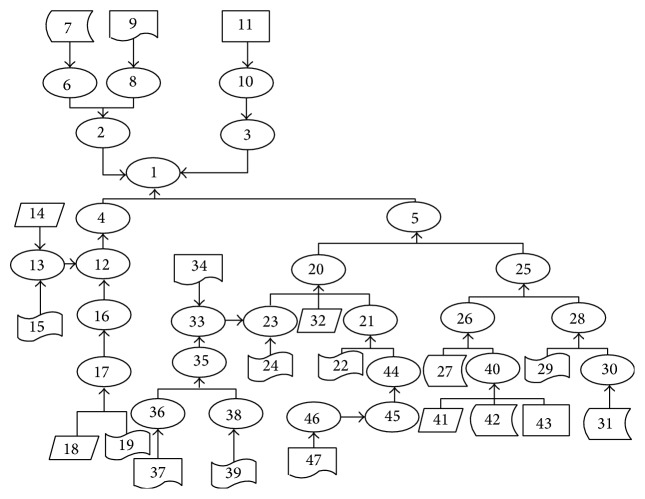
The CDMBE graph of “Witness for the Prosecution.”

From the description of the case in the novel “Witness for the Prosecution,” we identified 47 key items from the case, as Table 1 shows. Firstly, the first five critical items can be added to the top-level CDMBE graph according to the constitutive requirements. Then the other evidences and assumptions can be added to the CDMBE graph step by step. When a key item is added to the graph, it is necessary to set its credibility and supportability. After the initialization, we can calculate the credibility of the final assumption by bottom-up order. The CDMBE graph is shown as in Figure 1.

In Table 1, ID is the node's identity. T means the type of node. A means assumption. E means evidence. V is Vole. F is French. FA means the identity of the node's father. S means supportability. R means syntagmatic relationship. J means conjunction. C means recombination. P means coupling. G means aggregation. N means reinforcement. IC means initial credibility. FC means final credibility.

### 4.3. Deducing the Court Trial Process

Using the CDMBE graph, the court trial process can be deduced by adding evidences and assumptions step by step, which can be used to analyze the changes caused by evidences. Firstly, a top-level graph is initialized by analyzing the constitutive requirements, including items 1 to 11. Then, the plaintiff adduces evidence groups 1 to 3. Finally, the defendant adduces evidence groups 4 to 7. The changes of the credibility of the final assumption are shown in Table 2.

Table 2 shows that the credibility of defendant guilt becomes larger with the prosecution's evidences' joining and becomes smaller with the defense's evidences' joining. It is consistent with the real-life legal reasoning. According to the effect of the prosecution's evidence, the evidence groups 6 and 8 are the two most important evidence groups identified by CDMBE and Fenton's model. As the change of the evidence, group 3 is identified as the key group by CDMBE, whereas group 2 is identified as the key group by Fenton model. In reality, if the defendant admits that he had killed a man, the probability of his guilt should be high.

## 5. Conclusion

In order to make the case description model more intuitive and more accurate to reason, we proposed the CDMBE, which defines assumption and evidence as basic elements, uses credibility and supportability to calculate the testimonial power, and uses syntagmatic relationships to reason the credibility of the final assumption. Experiments show that the CDMBE model is easy to be understood, by which users can clarify their thoughts. At the same time, the model has a strict calculability and can reason legal argument quantitatively. And its analysis results are similar to that of Fenton's object-oriented Bayesian model and are accordant with reality.

## Figures and Tables

**Table 1:** The key items of “Witness for the Prosecution.”

ID	T	Description	FA	S	R	IC	FC
1	A	V murdered F			J	0	0.11
2	A	V had a full capacity for criminal responsibility	1	1	C	0	1
3	A	Right to life of F	1	1	P	0	1
4	A	V had criminal motive and purpose	1	1	P	0	0.91
5	A	V killed F	1	1	C	0	0.11
6	A	V was normal	2	1	P	0	1
7	E	V's psychiatric expertise	6	1		1	1
8	A	V was at the criminal legal age	2	1	P	0	1
9	E	V's age certification	8	1		1	1
10	A	F died	3	1	P	0	1
11	E	The body of F	10	1		1	1
12	A	V had a motive for money	4	1	G	0	0.91
13	A	V was poor	12	0.9	G	0	0.96
14	E	V claimed to be poor	13	0.9		1	1
15	E	Others testified that V was poor	13	0.9		1	1
16	A	V had a motive for money	12	0.9	P	0	0.89
17	A	V consulted a line trip abroad	16	0.9	N	0	0.99
18	E	V's testimony	17	0.9		1	1
19	E	Travel agency employee's testimony	17	1		1	1
20	A	V was at the crime scene	5	1	G	0	0.11
21	A	V admitted that he killed F	20	1	P	0	0.26
22	E	Christine's testimony	21	0.95		1	1
23	A	Servant heard the talking between V and F	20	1	P	0	0.21
24	E	Servant heard the joking between V and F	23	0.52		1	1
25	A	F died of a hit from the back of her head by V	5	1	G	0	0.45
26	A	The bloodstain on V's clothes was F's	25	1	P	0	0.24
27	E	The blood type on the clothes was the same as F's	26	0.93		1	1
28	A	F died of a hit from the back of her head	25	0.45	G	0	0.99
29	E	Police Herne proved a fatal hit	28	0.9		1	1
30	A	French's head was hit by blunt	28	1	P	0	1
31	E	The medical report	30	1		1	1
32	E	V reported that he was not at the crime scene	20	−0.1		1	1
33	A	Servant lied	23	−0.9	G	0	0.93
34	E	The change of the will made V become the biggest beneficiary	33	0.8		1	1
35	A	Servant did not hear the joking	33	0.95	G	0	0.96
36	A	Servant was disabled in hearing	35	0.9	P	0	1
37	E	Servant applied for hearing aid	36	1		1	1
38	A	The bedroom door was closed	35	0.9	P	0	1
39	E	Servant proved that the bedroom door was closed at that time	38	1		1	1
40	A	The bloodstain on V's clothes was his	26	−0.8	G	0	0.95
41	E	V's testimony: he injured while cutting bread	40	0.5		1	1
42	E	V's blood type was the same as F's	40	0.6		1	1
43	E	V had a scar on his hand	40	0.95		1	1
44	A	Christine lied about V confession to him and the time of coming back	21	−0.9	P	0	0.82
45	A	Christine said in a letter that he would perjure and elope with lover	44	0.9	P	0	0.9
46	A	All of the letters were written to his lover overseas	45	0.9	P	0	1
47	E	A letter from Christine to his lover overseas	46	1		1	1

**Table 2:** The influence of evidences on final credibility.

The order of proof	CDMBE	Fenton model
Prosecution evidence presented		
(1) Motive evidence: maid testifies Vole was present: 12–19	0.45	0.526
(2) Blood matches F evidence: items 25–31	0.60	0.865
(3) Romaine testifies Vole admitted guilt: items 20–24	0.91	0.966

Defense evidence presented		
(4) Vole testifies he was not present: item 32	0.86	0.969
(5) Maid evidence accuracy: items 33–39	0.82	0.913
(6) Blood matches Vole: items 40–42	0.55	0.644
(7) Vole shows scar: item 43	0.45	0.404
(8) Letters as evidence: items 44–47	0.11	0.149
